# Inconsistent Effects of Parietal α-tACS on Pseudoneglect across Two Experiments: A Failed Internal Replication

**DOI:** 10.3389/fpsyg.2017.00952

**Published:** 2017-06-08

**Authors:** Domenica Veniero, Christopher S.Y. Benwell, Merle M. Ahrens, Gregor Thut

**Affiliations:** ^1^Institute of Neuroscience and Psychology, University of GlasgowGlasgow, United Kingdom; ^2^School of Psychology, University of GlasgowGlasgow, United Kingdom

**Keywords:** tACS, tDCS, landmark task, replication, tES reliability

## Abstract

Transcranial electrical stimulation (tES) is being investigated as an experimental and clinical interventional technique in human participants. While promising, important limitations have been identified, including weak effect sizes and high inter- and intra-individual variability of outcomes. Here, we compared two “inhibitory” tES-techniques with supposedly different mechanisms of action as to their effects on performance in a visuospatial attention task, and report on a direct replication attempt. In two experiments, 2 × 20 healthy participants underwent tES in three separate sessions testing different protocols (10 min stimulation each) with a montage targeting right parietal cortex (right parietal–left frontal, electrode-sizes: 3cm × 3cm–7 cm × 5 cm), while performing a perceptual line bisection (landmark) task. The tES-protocols were compared as to their ability to modulate pseudoneglect (thought to be under right hemispheric control). In experiment 1, sham-tES was compared to transcranial alternating current stimulation at alpha frequency (10 Hz; α-tACS) (expected to entrain “inhibitory” alpha oscillations) and to cathodal transcranial direct current stimulation (c-tDCS) (shown to suppress neuronal spiking activity). In experiment 2, we attempted to replicate the findings of experiment 1, and establish frequency-specificity by adding a 45 Hz-tACS condition to α-tACS and sham. In experiment 1, right parietal α-tACS led to the expected changes in spatial attention bias, namely a rightward shift in subjective midpoint estimation (relative to sham). However, this was not confirmed in experiment 2 and in the complete sample. Right parietal c-tDCS and 45 Hz-tACS had no effect. These results highlight the importance of replication studies, adequate statistical power and optimizing tES-interventions for establishing the robustness and reliability of electrical stimulation effects, and best practice.

## Introduction

Transcranial electrical stimulation (tES) is an umbrella term for non-invasive brain stimulation techniques that involve the application of weak electrical currents to the brain. These techniques are increasingly being employed as tools in cognitive neuroscience and investigated as to their potential for novel clinical interventions in various neurological and psychiatric disorders(Berlim et al., 2013; Baeken et al., 2016; Lefaucheur, 2016). However, while some results provide evidence of cognitive enhancement and therapeutic benefit through tES across a range of cognitive domains (see Santarnecchi et al., 2015 for a review of neuroenhancement in healthy samples and Brunoni et al., 2012; Fregni et al., 2015 for discussions of clinical research), there remains little understanding of tES mechanisms of action and questions have been raised about the reliability of its effects (Bestmann et al., 2015; Horvath et al., 2015a,b; Parkin et al., 2015). Large variability in tES-outcome has been shown within and across studies. This variability is likely to be explained by the large number of factors influencing outcome (Fertonani and Miniussi, 2016), including current strength/density (Teo et al., 2011; Moos et al., 2012; Bastani and Jaberzadeh, 2013; Batsikadze et al., 2013; Hoy et al., 2013; Benwell et al., 2015; Ho et al., 2016), electrode montage (Moliadze et al., 2010; Sehm et al., 2013; Scheldrup et al., 2014; Mehta et al., 2015), stimulation duration (Nitsche and Paulus, 2000; Stagg and Nitsche, 2011), stimulation frequency (Kanai et al., 2010; Feurra et al., 2011; Brignani et al., 2013; Wach et al., 2013; Cabral-Calderin et al., 2016), timing of stimulation relative to task engagement (Pirulli et al., 2013; Scheldrup et al., 2014; Bortoletto et al., 2015; Cabral-Calderin et al., 2016), baseline trait/task performance levels of participants (Dockery et al., 2009; Tseng et al., 2012; Hsu et al., 2014; Sarkar et al., 2014; Benwell et al., 2015; Learmonth et al., 2015; Li et al., 2015b; London and Slagter, 2015), task demands (Li et al., 2015a; Roe et al., 2016) and the initial excitatory/oscillatory state of the stimulated region (Feurra et al., 2013). In addition to the difficulty of understanding all of the potential contributors to tES outcome, its effects tend to be relatively small and difficult to replicate (Walsh, 2013).

Here, we sought to directly compare two different tES techniques as to their efficacy to change behavior within the same experimental setting (same participant, task, montage) and to establish reproducibility in a follow-up experiment. In the first experiment, we compared cathodal tDCS (c-tDCS) and tACS at 10 Hz, i.e., in the alpha-band (8–12Hz) (α-tACS) against sham tES as to their ability to modulate visuospatial attention. We chose these protocols because both have been associated with “inhibitory” effects on attention tasks, despite their supposedly different mechanisms of action, namely a decrease of cortical excitability through an alteration of the resting neuronal membrane threshold for c-tDCS (Bindman et al., 1964; Nitsche and Paulus, 2000; Nitsche et al., 2005) or the synchronization of oscillatory activity in the alpha-band for α-tACS (Zaehle et al., 2010; Antal and Paulus, 2013; Herrmann et al., 2013; Helfrich et al., 2014; Veniero et al., 2015). Alpha-band activity has been shown to inversely relate to visual cortex excitability (Romei et al., 2008) in line with its proposed inhibitory role (Klimesch et al., 2007; Jensen and Mazaheri, 2010).

In order to assess tES outcome on attention processes, we employed a perceptual variant of line bisection (the landmark task: Milner et al., 1992; McCourt and Olafson, 1997) measuring spatial attentional bias [usually biased toward the left in heathy participants, c.f. pseudoneglect (Bowers and Heilman, 1980; Jewell and McCourt, 2000)], and which is thought to be driven by a right hemisphere (RH) dominance (for neuroimaging evidence, see Fink et al., 2000; Weiss et al., 2000; Foxe et al., 2003; Cicek et al., 2009; Thiebaut de Schotten et al., 2011; Benwell et al., 2014a). We chose the landmark task because of its high test–retest reliability (Benwell et al., 2013b, 2015), and because simple experimental manipulations (e.g., time-on-task) can induce reproducible short-term changes in line bisection bias (Manly et al., 2005; Dufour et al., 2007; Benwell et al., 2013a). We reasoned that a combination of high test–retest reliability and amenability to experimental manipulation constitutes an important precondition when probing for potentially weak tES effects. Specifically, we hypothesized that both right parietal c-tDCS and right parietal α-tACS would cause an inhibition of right hemispheric attention processes and thus result in a similar behavioral outcome on attentional bias in the landmark task, namely canceling out the systematic group-level leftward bias (i.e., pseudoneglect) (Benwell et al., 2013a,b, 2014a,b). This is motivated by previous tDCS research in healthy participants showing that right parietal c-tDCS drives a rightward shift in subjective midpoint estimation (Giglia et al., 2011; Wright and Krekelberg, 2014; Benwell et al., 2015). Right parietal α-tACS, on the other hand, may influence attentional bias by up-regulating parieto-occipital alpha power (see Thut et al., 2006; Newman et al., 2013, 2017 implicating EEG alpha activity in visuospatial bias) and thereby downregulate RH contribution to landmark task processing and ameliorate pseudoneglect.

In the second experiment, we aimed to reproduce effects of active versus sham tES observed in the first experiment in an independent sample of participants. We also added an extra tACS condition (45 Hz, γ-tACS) to test for frequency-specificity of outcome, as γ-tACS is not expected to induce an inhibitory effect on attentional processes.

## Materials and Methods

### Participants

A total of 40 right-handed volunteers participated after having given their written informed consent. In the first experiment, 20 participants were recruited. One participant was excluded due to poor task performance [curve fitting procedures showed a low goodness of fit in one of the conditions (*R*^2^ = 0.5), see analysis below for details]. Data from 19 participants were therefore considered for the final analysis of experiment 1 (12 female, mean age: 23.6, *SD* = 3.8). In the second experiment, an independent group of 20 participants was enrolled (10 female, mean age: 23.8, *SD* = 2.8).

All participants had normal or corrected-to-normal vision and had no contraindication to brain stimulation according to the screening questionnaire described in Rossi et al. (2009) or reported any neurological, psychiatric, or other relevant medical problems. The protocol was performed in accordance with ethical standards and was approved by the Ethics Committee of the College of Science and Engineering (University of Glasgow).

### Task and Stimuli

In both experiments, participants performed a modified version of the landmark task (Milner et al., 1992; Benwell et al., 2015) in which pre-transected black and white lines of 100% Michelson contrast are presented on a gray background (luminance = 179, hue = 160). Lines measured 24.3 cm by 0.5 cm and subtended 19.67° by 0.40° of visual angle at a viewing distance of 70 cm. Lines were transected at 17 different points ranging symmetrically ±4% of absolute line length from veridical center. This represented a range of -0.8 to 0.8° of visual angle relative to veridical center. Stimuli were presented on a CRT monitor with a 1280 pixel × 1024 pixel resolution using E-prime (Psychology Software Tools, Pittsburgh, PA, United States).

**FIGURE 1:**
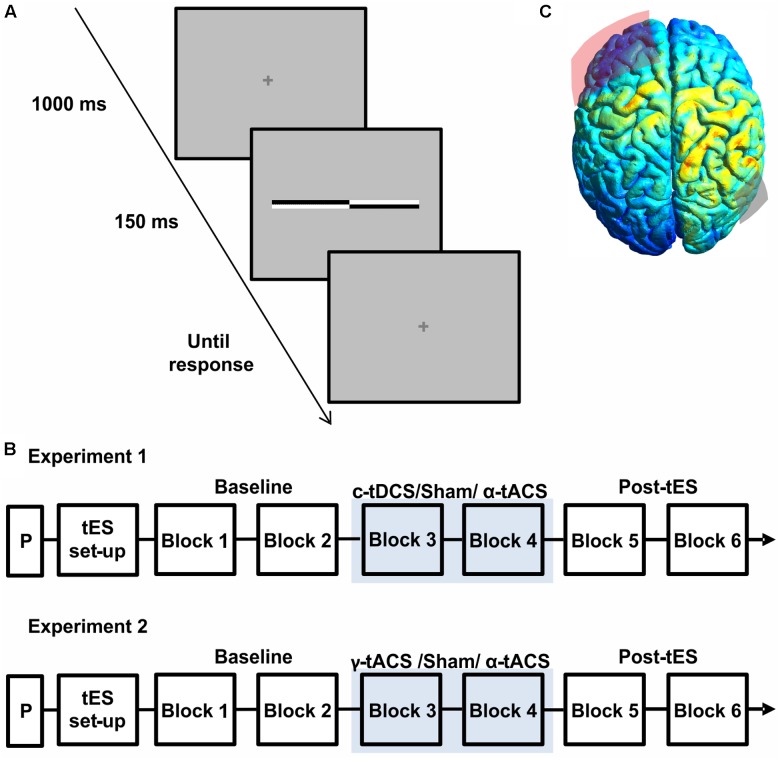
**(A)** Schematic representation of a single trial structure. A fixation cross was presented for 1000 ms, followed by a transected line flashed for 150 ms. The fixation cross reappeared for the response time period. **(B)** Schematic representation of the experimental procedure of both experiments. On each day, after a practice session (P) and transcranial electrical stimulation (tES) set up, participants were asked to complete six blocks of the landmark task. During block 3 and block 4 participants received active tES or sham in a counter-balanced order across days. **(C)** Results of electric field distribution modeling are shown together with the electrode position (F3 in red, P6 in gray). The electric field strength is scaled from 0 (blue) to maximum (red). The simulation indicates diffuse activation within the right parietal cortex together with a more restricted activation of a left frontal region.

Each trial started with a central fixation cross (0.4 × 0.4° visual angle) displayed for 1 s, followed by the presentation of a transected line for 150 ms (**Figure 1A**). The transection mark was always aligned with the fixation cross, therefore preventing use of the fixation cross as a reference point for bisection judgments. The fixation cross then reappeared for the response period, during which participants indicated as fast and accurately as possible which end of the line the transection mark had appeared closest to, by pressing either a left or right response key with the index or middle finger of the right hand. Participants were instructed to keep their gaze on the fixation cross. The next trial started as soon as the response was made (**Figure 1A**). The location of the transector point was randomly selected in each trial.

### Transcranial Electrical Stimulation

Transcranial electrical stimulation was delivered through a battery-driven, constant current stimulator (NeuroConn GmbH, Germany) using two surface electrodes placed in saline-soaked sponges kept in place by an elastic net. To optimize skin/electrode impedance a thin layer of electroconductive gel was applied to the sponges (Horvath et al., 2014; Fertonani et al., 2015) (for similar practices see, e.g., also Pirulli et al., 2013; Tecchio et al., 2013; Romero Lauro et al., 2014; Li et al., 2015a). Impedance values were maintained below 5 kΩ. The electrodes positions were individually located in accordance with the International 10–20 EEG system: the target electrode (3 cm × 3 cm) was placed over the right parietal cortex (P6), whereas the frontal electrode (7 cm × 5 cm) was centered over F3. The size of the frontal electrode was chosen to reduce current density and limit stimulation effects under its surface (Nitsche et al., 2007) relative to the parietal electrode, and the slightly more superior position, relative to the frequently used supra-orbital site (FP1), was chosen to limit phosphene perception. The F3 location was determined based on a pilot. In 3 participants (none of whom included in experiment 1 or 2), we assessed the amount of visual sensations induced by α-tACS under slightly different montages (moving the frontal electrode from FP1 to F3 while keeping the parietal electrode at P6). All participants reported visual sensations with the frontal electrode over FP1. When moving the frontal electrode to F3 (without lowering the stimulation intensity), phosphene perception was absent in these 3 participants. In the participants of the main experiments, phosphene perception was probed through a questionnaire asking about tES-induced sensation, which was given at the end of each session (see Procedure and Results section). Unilateral parietal stimulation (P6) was chosen (as opposed to bilateral parietal stimulation applied in some previous tDCS-studies, e.g., P6–P7) to be able to selectively interfere with right parietal cortex during both c-tDCS and α-tACS (bi-parietal α-tACS would have affected both hemispheres in the same way).

For all conditions, stimulation intensity was set at 1 mA, yielding an average current density of 0.11 and 0.03 mA/cm^2^ for the parietal and frontal electrode, respectively. The anatomical target of stimulation was verified by modeling the electric current field distribution induced with 1 mA intensity and the above montage using SimNIBS^1^ (Thielscher et al., 2015), adopting the standard head model provided by the software.

Each participant underwent three stimulation conditions, which consisted of c-tDCS, α-tACS and sham tES in experiment 1, and α-tACS, γ-tACS and sham tES in experiment 2. For tDCS, the polarity of the target electrode (P6) was cathodal (c-tDCS). For tACS, a sinusoidal electrical current waveform with no DC offset was delivered at 10 Hz (α-tACS) or 45 Hz (γ-tACS). The total stimulation time was 10 min, including 30 s ramping up/down except for the sham condition, during which the stimulator was turned off after 30 s (10 s ramping up/down).

For both experiments, the three sessions were separated by at least 48 h with the order of tES protocols counter-balanced across participants to control for learning and carry-over effects.

### Procedure

Participants were seated in a comfortable armchair in a dimly lit room. The viewing distance (70 cm) was kept constant throughout the session using a chin rest. Each session started with a practice block (**Figure 1B**) including two trials for each transector location for a total of 34 trials. Participants were then prepared for the stimulation (tES setup, **Figure 1B**). During each session (experiment 1: c-tDCS, α-tACS and Sham; experiment 2: α-tACS, γ-tACS, and Sham), participants were asked to complete six blocks of landmark task performance, consisting of 136 trials (8 trials for 17 transector point), lasting around 5 min each. As show in **Figure 1B**, stimulation was applied during block 3 and 4, whereas the first and last two blocks were performed without stimulation and served as a baseline (blocks 1–2) and to test for potential after-effects (blocks 5–6) (**Figure 1B**). At the end of the blocks, participants were asked to complete a questionnaire implemented to evaluate sensations experienced during tES including phosphenes (Fertonani and Miniussi, 2016) on a scale of 0 (not experienced) to 4 (experienced very strongly). Data from this questionnaire were analyzed using a Friedman ANOVA to assess side-effects across sessions. At the end of all sessions, participants were asked to guess on which day they had received sham stimulation.

### Analysis

To obtain an objective measure of perceived line midpoint for each block and session in each subject, psychometric functions (PFs) were derived using the method of constant stimuli. The dependent measure was the percentage of trials on which participants indicated that the transector had appeared closer to the left end of the line. Non-linear least-squares regression was used to fit a cumulative logistic function to the data for each block and stimulation condition in each subject. The cumulative logistic function is described by the equation:

f(μ,x,w)=1/(1+exp⁡((x−μ)/−w))

where *x* represents the transection locations, μ corresponds to the estimated transector location with 50:50% “left”:“right” response rate and *w* is the estimated width of the psychometric curve (both measured in pixels on the *x*-axis). The 50% location is also known as the point of subjective equality (PSE) and represents an objective measure of perceived line midpoint. The width of the fitted PF provides a measure of the precision of the participants’ line midpoint judgments (visual discrimination sensitivity).

As PSE represented our dependent (outcome) measure of interest, we assessed its test–retest reliability within participants using robust correlation analysis between values obtained during the baseline blocks (average of blocks 1–2) in each session, i.e., across 3 days. This analysis was performed using Spearman’s rho and Shepherd’s Pi (Schwarzkopf et al., 2012). tES effects on PSE were analyzed using a repeated measure analysis of variance (ANOVA) testing the factors tES (Experiment 1: c-tDCS vs. α-tACS vs. Sham; Experiment 2: α-tACS vs. γ-tACS vs. Sham) and Block (B1–B6).

Finally, in order to quantify whether any effects observed in experiment 1 could be successfully replicated in experiment 2, we calculated the Bayes factor (B) which allows a quantification of how strong the evidence is for the alternative or the null hypothesis, here the presence or absence of stimulation effect. Despite the fact that evidence is continuous, *B* < 1/3 can be considered as strong evidence in favor of the null hypothesis, *B* > 3 as strong evidence in favor of the alternative hypothesis, whereas 1/3 < *B* < 3 indicates data insensitivity (support for neither hypothesis) (Dienes, 2014; Verhagen and Wagenmakers, 2014). We modeled the plausibility of different effects by a half normal distribution (Dienes and Mclatchie, 2017) and corrected for the degrees of freedom as in Dienes (2014).

## Results

### Electric Field Modeling

**Figure 1C** shows the results of the current distribution modeling. The electric field strength peaked within the right parietal cortex, suggesting that the chosen montage most strongly stimulates our target site. The modeling also indicated stimulation of a more spatially restricted part of the left frontal lobe, albeit at a weaker current.

## Experiment 1

### Sensations during tES

Transcranial electrical stimulation did not cause any of the participants to experience adverse effects. The results of the questionnaire indicated a low mean difference in sensation scoring when comparing the active with the sham condition (active tES minus sham ∼0.2). Three participants reported visual sensations at the beginning of α-tACS. A Friedman’s test used to compare the strength of the sensations in each session revealed no significant difference (*p* = 0.88), indicating that all protocols were similar in associated (low) discomfort. Eight participants correctly identified the sham session (42%). A chi square goodness of fit run to compare the percentage of correct guesses (42% = 8/11) with the expected (chance) occurrence (33%: 6/13) revealed no significant deviation from the expected value [*X*^2^(1) = 0.97; *p* = 0.32], thus confirming that the percentage of participants correctly identifying the sham condition was not different from chance.

### Spatial Attention Bias: Test–Retest Reliability across Baseline Blocks

**FIGURE 2:**
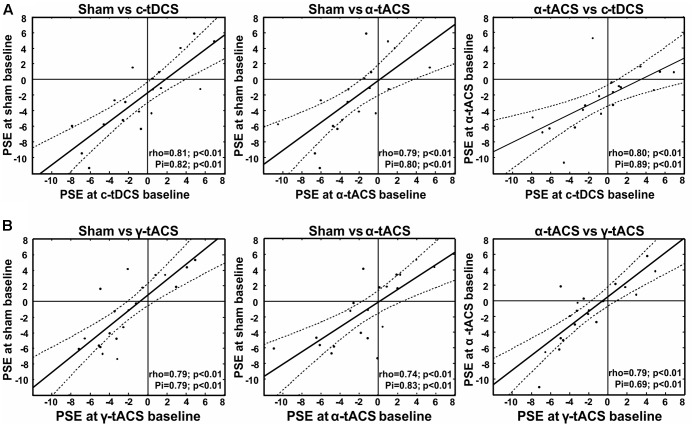
Test re-test reliability of spatial bias in perceptual line bisection (point of subjective equality, PSE) for experiment 1 **(A)** and experiment 2 **(B)**. Test–retest reliability is significant across three measurement points collected before tES (baseline) at three different days, as indicated by both Spearman’s rho and Shepherd’s Pi values (see lower right corner of each graph). *x*- and *y*-values represent the distance from the veridical center in pixels (dashed lines represent 95% confidence intervals).

To inform the interpretation of potential tES effects on our variable of interest (subjective midpoint in line bisection/attentional bias), we first analyzed the data of the baseline blocks before tES (blocks 1–2) in terms of an overall deviation of subjective midpoint from the veridical midline (i.e., pseudoneglect), as well as its test–retest reliability within participants. We found a leftward bias at the group-level (pseudoneglect) across the three sessions (all baseline data collapsed) [one-sample *t*-test against zero, *t*(18) = -2.09, *p* = 0.05] and good test–retest reliability of individual baseline bias across the three (i.e., α-tACS, c-tDCS and sham) sessions (see **Figure 2A** for scatterplots and correlation analysis results). This confirms previous results (for pseudoneglect see, e.g., Jewell and McCourt, 2000 and for test–retest reliability see Benwell et al., 2013b, 2015; Varnava et al., 2013). It is of interest to note that while there is a leftward bias at the group level, and a rather low intra-individual variability across sessions (standard deviation ranged from 0.4 to 3.8 pixels), the inter-individual variability is relatively large (min = -11.3 pixels, max = +6.9, see **Figure 2A**), also in line with previous findings (Jewell and McCourt, 2000; Benwell et al., 2013b; Szczepanski and Kastner, 2013).

### Spatial Attention Bias: Effects of α-tACS and c-tDCS

**FIGURE 3:**
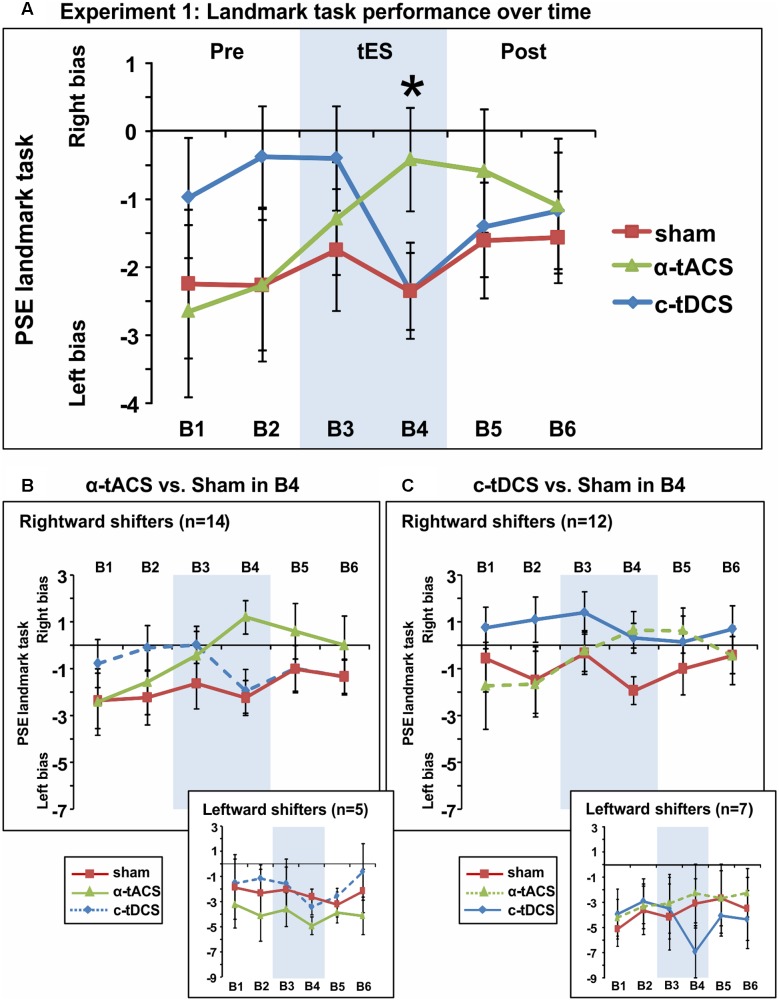
Spatial bias in perceptual line bisection (PSE during landmark task performance) over time (six blocks) in three experimental sessions varying in terms of the tES protocol used (experiment 1). tES was applied with a right parietal – left frontal montage for 10 min using either α-tACS, c-tDCS or sham tES during blocks 3–4 (B3–4, shaded area). **(A)** Overall group mean PSE values (19 participants, within-subject design) as a function of blocks (B1–6) and tES (line plots). During the second block of tES (block 4), α-tACS significantly differed from sham and c-tDCS. The asterisk indicates a significant difference between Sham and α-tACS (*p* < 0.05). **(B)** Performance split according to “rightward shifters” (upper main graph, 14/19 participants) and “leftward shifters” (lower inset, 5/19 participants) during α-tACS (as compared to sham in block 4). The pattern of results is consistent with α-tACS having shifted spatial bias rightward (reduced pseudoneglect) in the majority of participants. **(C)** Performance split according to “rightward shifters” (upper main graph, 12/19 participants) and “leftward shifters” (lower inset, 7/19 participants) during c-tDCS (as compared to sham in block 4). No effects of active stimulation (c-tDCS vs. sham) are discernible in this condition and population. The error bars represent 95% confidence interval corrected for a within subjects design (Cousineau, 2005).

We next analyzed the influence of tES on attentional bias (see **Figure 3A** for the group-averaged data per condition). The ANOVA revealed an interaction between the factors tES (α-tACS vs. c-tDCS vs. Sham) and Blocks (B1–6) [*F*(10,180) = 2.51, *p* = 0.008, $\eta_{p}^{2}$ = 0.12] suggesting that active and sham tES differently affected subjective midline judgment over time. No main effects were observed [tES: *F*(2,36) = 0.96, *p* = 0.39, $\eta_{p}^{2}$ = 0.05, Block: *F*(5,90) = 1.17, *p* = 0.33, $\eta_{p}^{2}$ = 0.06]. Breaking down the interaction revealed a significant effect of tES (α-tACS vs. c-tDCS vs. Sham) in block 4 [*F*(2,36) = 3.81, *p* = 0.032, $\eta_{p}^{2}$ = 0.17], i.e., during the second half of the 10 min of tES, while there was no significant tES effect in any other block [*F*(2,36) = 0.13–2.49, *p* = 0.88–0.10]. By block 4, α-tACS had shifted spatial attention rightward (see **Figure 3A**, PSE significantly less negative than in the other two conditions), both in comparison to Sham [B4: *F*(1,18) = 7.00, *p* = 0.016, *d* = 0.38] and c-tDCS [B4: *F*(1,18) = 4.79, *p* = 0.042, *d* = 0.36]. c-tDCS did not differ from Sham [*F*(1,18) = 0.0001, *p* = 0.99, *d* = 0.00] at block 4. Overall, this suggests that as expected, α-tACS over right parietal cortex may have redirected attention to the ipsilateral, right hemifield. In contrast to our expectations, c-tDCS did not seem to affect task performance.

In order to further explore the effect of tES and to test whether subgroups of tES-responders (either for α-tACS or c-tDCS) could be identified, we split participants for each stimulation condition into “rightward shifters” (responders as predicted by our hypothesis) and “leftward shifters” (non-responders) according to their difference in PSE between the active and sham conditions at block 4 (B4 α-tACS or B4 c-tDCS compared to B4 sham). Calculating the number of responders for α-tACS revealed a subgroup of 14 “rightward shifters” (illustrated in **Figure 3B**, upper large panel) and 5 “leftward shifters” (**Figure 3B**, lower small panel). The majority of participants responded over time to α-tACS with a progressive rightward shift away from sham performance (**Figure 3B**, upper large panel, green versus red line). Splitting the data in the same way for c-tDCS (B4 c-tDCS vs. B4 sham) revealed a different picture. While 12 versus 7 participants were more “rightward oriented” in block 4 (see **Figure 3C**, upper large panel versus lower small panel), there was no sign of a progressive rightward shift during tDCS away from sham performance over time as differences already pre-existed prior to stimulation blocks. By extension, there is therefore no evidence even for a subgroup of participants responding as expected to tDCS. This further confirms that c-tDCS did not affect task performance.

Overall, experiment 1 therefore suggested that α-tACS had shifted spatial attention bias in the majority of participants (*n* = 14, 74%) in the expected direction, while there was no evidence of a c-tDCS effect. The average amplitude of the tACS-induced change in responders (i.e., the amplitude of the rightward shift) was in the order of the within-subject variability that can be observed across sessions (possibly due to state-changes). In line with this observation, we indeed observed a small effect size (Cohen’s *d* = 0.36–0.38). We therefore aimed to replicate and extend the result of experiment 1 in an independent sample of participants using α-tACS, with γ-tACS and sham-tES as control conditions. We also reasoned that adding 20 participants should strengthen the α-tACS effects in the whole sample if present (i.e., when the α-tACS and sham-tES conditions of experiment 1 and 2 are combined).

## Experiment 2

### Sensations during tES

As for experiment 1, tES did not cause any of the participants to experience adverse effects and the scores of the sensation questionnaires only minimally differed between active and sham tES conditions (active tES minus sham ∼0.1). The strength of the sensations in each session showed no significant difference (*p* = 0.39), with 35% of participants (7 out of 20) correctly identifying the sham session, which corresponds to chance-level, i.e., the number of participants who would correctly guess by chance. Five participants reported visual sensations at the beginning of α-tACS.

### Spatial Attention Bias: Test–Retest Reliability across Baseline Blocks

As for experiment 1, we first tested whether we could find a leftward bias in subjective midpoint at the group-level (pseudoneglect) across the three baseline sessions (all baseline data collapsed) and whether we could replicate the high test–retest reliability of individual baseline bias across the three sessions. A one-sample *t*-test (against zero) again revealed a leftward bias [*t*(19) = -2, *p* = 0.06], and the correlation analysis confirmed consistency of PSE within participants as shown in **Figure 2B**.

### Spatial Attention Bias: No Replication of α-tACS Effects

**FIGURE 4:**
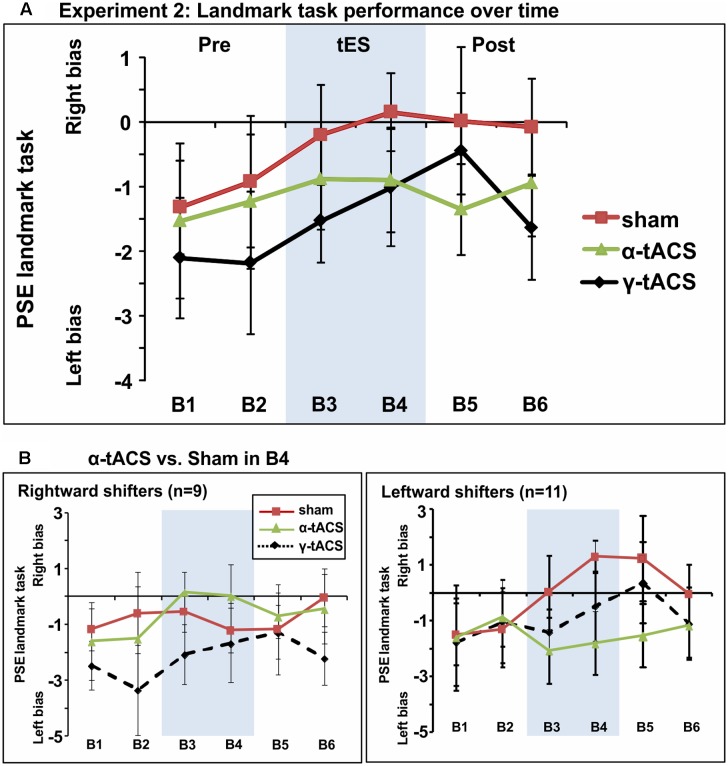
**(A)** Spatial bias in perceptual line bisection (PSE during landmark task performance) over time (six blocks) as a function of tES condition in experiment 2. The montage, intensity and total stimulation duration were identical to experiment 1. tES conditions consisted of α-tACS, 45 Hz (γ-tACS) or sham tES applied during blocks 3–4 (B3-4, shaded area). PSE values (20 participants, within-subject design) showed no significant difference across conditions in this population. Error bars represent 95% confidence interval corrected for a within subjects design. **(B)** Performance split according to “rightward shifters” (right panel, 9/20 participants) and “leftward shifters” (left panel, 11/20 participants) during α-tACS (as compared to sham in block 4). Unlike experiment 1, α-tACS reduced pseudoneglect in 45% of the population (inconsistent, random distribution).

**Figure 4A** shows group-averaged data for each condition. The main ANOVA performed to assess tES effects (α-tACS vs. γ-tACS vs. Sham) over the six blocks (B1–6) revealed a significant main effect of tES [*F*(2,38) = 3.44, *p* = 0.04, $\eta_{p}^{2}$ = 0.15], explained by generally higher PSE values in the Sham compared to γ-tACS condition [*F*(1,19) = 6.05, *p* = 0.02, $\eta_{p}^{2}$ = 0.24], but no main effect of Block [*F*(5,95) = 1.75, *p* = 1.13, $\eta_{p}^{2}$ = 0.08]. Most importantly, the analysis revealed that there was no significant tES × Block interaction [*F*(10,190) = 0.73, *p* = 0.69, $\eta_{p}^{2}$ = 0.04], suggesting that we could not replicate the α-tACS induced rightward shift found in experiment 1. The lack of a significant interaction implied that in this second sample, α-tACS was unable to induce any modulation of PSE relative to sham at any time point. In order to quantify the number of participants showing the α-tACS-induced effect found in experiment 1, we again split our group into rightward and leftward attention shifters according to PSE values in block 4 (B4 α-tACS vs. B4 sham). As shown in **Figure 4B**, α-tACS modulated the attentional bias in the expected direction in only 9 out of the 20 participants, whereas the remaining 11 were classified as leftward shifters, further confirming the inconsistency of results in this experiment.

In order to quantify more precisely the outcome of our replication attempt, we calculated the Bayes factor on the data from experiment 2. Our null hypothesis was that the effect of α-tACS and sham are equivalent and therefore the results of experiment 2 represent a failed replication of experiment 1. Our alternative hypothesis (H1) was that tACS stimulation differs from the sham stimulation, by an amount and direction informed by the first experiment. Hence, our measure of interest (α-tACS effect) was the magnitude of the α-tACS induced shift in PSE (B4 α-tACS minus B1 α-tACS) relative to the PSE shift in the sham condition (B4 Sham minus B1 Sham). The predictions of H1 were modeled as half-normal distribution (B_H_) with a SD equal to the effect obtained in the first experiment (rightward shift in the α-tACS relative to the sham condition = 2.22 pixels). The analysis revealed that *B* is <1[*B*_H(0,2.22)_ = 0.26], thus providing substantial evidence in favor of the null hypothesis indicating a failed replication.

## Data of Experiment 1 and 2 Collapsed

### Spatial Attention Bias: Effect of α-tACS in Experiment 1 Dissolves in the Whole Sample

**FIGURE 5:**
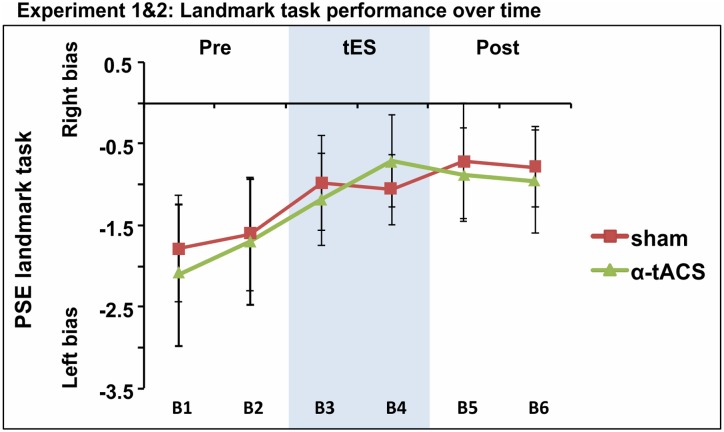
Spatial bias in perceptual line bisection (PSE) over time (six blocks) as a function of tES condition when data from experiment 1 and 2 were collapsed over common conditions (Sham, α-tACS). PSE values showed no difference across conditions in the overall population (39 participants). Error bars represent 95% confidence interval corrected for a within subjects design.

Based on the idea that inconsistency in tES outcome might be partly explained by low statistical power, given the small to intermediate effect sizes usually reported (see experiment 1 Cohen’s *d* and Horvath et al., 2016; Minarik et al., 2016; Schutter and Wischnewski, 2016), we collapsed the PSE data of both experiments for the two common conditions. The whole sample therefore included 39 participants who underwent α-tACS and sham stimulation. The repeated-measures ANOVA testing factors tES (Sham, α-tACS) and Blocks (B1–6) revealed no significant tES × Block interaction [*F*(5,190) = 0.24, *p* = 0.94, $\eta_{p}^{2}$ = 0.00; **Figure 5**]. In line with previous studies, we found a progressive left- to rightward shifts in line bisection judgment over time [time-on-task effect: main effect of Block: *F*(5,190) = 3.37, *p* = 0.006, $\eta_{p}^{2}$ = 0.08], regardless of the stimulation condition. By block 3, PSE was significantly less negative when compared to the first baseline block [mean B1 PSE = -1.9, mean B3 PSE = -1.08, mean B4 PSE = -0.89, mean B5 PSE = -0.80, mean B6 PSE = -0.88, all *F*(1,38) ≥ 3.99, all *p* ≤ 0.05, *d* ≥ 0.21].

To investigate whether participants’ spatial bias at baseline may have affected tES outcome (e.g., may have contributed to the negative findings), participants were split into two subgroups according to the median of PSE values at baseline (each individual value was calculated as the average of block 1 across α-tACS and Sham). A 2 × 6 × 2 mixed-design ANOVA was performed with tES (α-tACS vs. sham) and Blocks (B1–6) as within-subject factors and PSE at baseline (above or below group median) as a between-subject factor. The ANOVA confirmed the progressive left- to rightward shift over time (main effect of Block [*F*(5,185) = 3.38, *p* = 0.006, $\eta_{p}^{2}$ = 0.08]), but revealed no effect of the initial spatial bias on tES outcome [no 3-way interaction of PSE at baseline × tES × Block: *F*(5,185) = 2.04, *p* = 0.07, $\eta_{p}^{2}$ = 0.005].

In the same vein, we tested whether our results could be explained by differences in discrimination sensitivity at baseline, defined as the average PF curve width in block 1 across the two sessions (as we have previously found tDCS-outcome on line bisection to depend on this measure, Benwell et al., 2015). Participants were therefore divided into two groups, one including those displaying baseline PF curve width below the median and one including those displaying width values above the median. tES effects on PSE were then tested through a 2 × 6 × 2 mixed design ANOVA with tES (α-tACS vs. Sham) and Blocks (B1–6) as within-subject factors and Curve Width at baseline (above or below group median) as a between-subject factor. The results again revealed a significant effect of block on PSE [*F*(10,170) = 2.53, *p* = 0.007, $\eta_{p}^{2}$ = 0.13] but no effect of the discrimination sensitivity at baseline on tES outcome [curve width × tES × Block: *F*(5,185) = 0.98, *p* = 0.43, $\eta_{p}^{2}$ = 0.02].

## Discussion

Across two experiments, we aimed to test to what extent previously reported parietal tES-effects on line bisection performance can be extended by introducing α-tACS and to compare its effects to c-tDCS, thereby providing new information on what could constitute promising stimulation parameters for interventions with existing, putatively “inhibitory” protocols. We also aimed to further address the tES replicability issue by attempting to replicate our own results in a second, independent sample. Our main findings were twofold. First, the results from experiment 1 showed no effect of c-tDCS on spatial attention bias, not replicating previously reported tDCS effects (Giglia et al., 2011; Benwell et al., 2015), whereas tACS delivered at 10 Hz caused a rightward shift in subjective midpoint estimation when compared to sham stimulation. This effect, however, was only present during stimulation and was characterized by a modest effect size. Second, when attempting to reproduce the α-tACS effect in an independent sample (experiment 2), we found no difference between α-tACS and sham stimulation. The null-effect was confirmed when testing the complete sample of participants (*n* = 39).

### Discrepancies with Previous tDCS Studies

Parietal tDCS has previously been reported to modulate visuospatial attention in a number of different tasks depending on polarity and stimulated hemisphere (Sparing et al., 2009; Loftus and Nicholls, 2012; Wright and Krekelberg, 2014; Benwell et al., 2015) (see Laczo et al., 2012; Li et al., 2015a for negative findings). Also using a line bisection task, Giglia et al. (2011) and Benwell et al. (2015) found a rightward shift in subjective midpoint estimation when cathodal tDCS was applied over the right parietal cortex. The discrepant findings in our study may be explained by differences in stimulation parameters. In both previous studies, tDCS was applied in a dual-parietal configuration, with the cathode over the right parietal cortex and the anode over the homologous left parietal area, while here we used unilateral parietal tDCS (with a second electrode over frontal cortex to selectively target right parietal cortex using the same montage also with α-tACS). However, Giglia et al. (2011) also tested unilateral right parietal c-tDCS with the anodal electrode over the contralateral orbit (i.e., similar to our electrode configuration) and found tDCS to reduce pseudoneglect, albeit with weaker effects compared to the bilateral montage. Differences in stimulation duration could also account for the discrepant findings, as in Giglia et al. (2011) and Benwell et al. (2015) total stimulation time was 20 min, whereas in the current study it was reduced to 10 min. Regarding tACS, no previous study has investigated tACS effects on attentional bias as measured by landmark task performance.

### Failed Replication Attempt: Points to Consider for Best Practice

Whilst the failed replication of previous tDCS effects may be accounted for by differences in task design, our failed direct internal replication of the tACS results is more unexpected.

A key point is that in the first experiment, we found statistically significant α-tACS effects, while the experiment was comparable in sample size to the majority of published tACS studies (we tested 19 participants; for previously used sample sizes see Schutter and Wischnewski, 2016, Table 1, average over 50 studies = 17 participants). It is therefore reasonable to assume that our first experiment would have been suitable for publication, although our results would have clearly overestimated the α-tACS effect if published stand-alone. These findings may cast doubts on the efficacy and reliability of tES (Wiethoff et al., 2014; Heroux et al., 2015; Horvath et al., 2015a) and/or point to a small to moderate effect size (see also Benwell et al., 2015; Hill et al., 2016; Mancuso et al., 2016; Minarik et al., 2016; Schutter and Wischnewski, 2016), which in combination with small sample sizes (Heroux et al., 2015; Grefkes and Fink, 2016; Minarik et al., 2016) increases the probability of false discoveries (Cappelleri et al., 1996; Ioannidis, 2005; Heroux et al., 2015; Vannorsdall et al., 2016) and the over- or underestimation of the effects of stimulation. It is possible that the α-tACS effect in experiment 1 may represent a false positive, as it was not confirmed in the second experiment (nor over the complete sample); or alternatively, our study may have been underpowered for revealing the true effect [although we merged the data from two experiments, *n* = 39; see Minarik et al. (2016) for a calculation of adequate sample size in tES studies]. However, while we would like to point out that our results may not be conclusive as to the efficacy of the present tES-protocols in modulating attentional bias, the inconsistent findings across our two studies clearly suggest that one should be cautious when interpreting results from single tES studies. Because testing large samples is not always realistic, internal replication attempts should be considered to establish tES efficacy before publication.

### Limitations in Design of Our Study and Suggestions for Improvements

It is conceivable that our overall unsuccessful attempt to modulate attention bias is based on the wrong choice of stimulation parameters, but also on the high variability in individual responses to brain stimulation (tES: Kuo et al., 2013; Lopez-Alonso et al., 2014; Wiethoff et al., 2014; TMS: Fratello et al., 2006; Hamada et al., 2013). The identification of factors that might contribute to this variability is crucial in order for them to be controlled for in future research (for a review on individual differences influencing tES outcome see Krause and Cohen Kadosh, 2014). Although identifying these factors and suboptimal design parameters is difficult, we point out below some potentially important changes to the overall ineffective stimulation approach we used here for consideration in future studies.

Evidence supporting the idea that tACS might engage neural activity through entrainment of endogenous oscillations has come from animal models (Ozen et al., 2010), computational models (Ali et al., 2013; Herrmann et al., 2016) and human studies (Helfrich et al., 2014; Neuling et al., 2015). According to this framework, matching the stimulation frequency to the individual natural frequency increases the efficacy of the stimulation by facilitating the emergence of entrainment (Pikovsky et al., 2003). In the present study, tACS frequency was not individually adjusted. It is therefore possible that stimulation was effective only in a small subsample (when individual alpha frequency matched the stimulation frequency of 10 Hz) potentially explaining the difference between experimental groups (i.e., in case of a higher number of individuals with an individual alpha frequency close to 10 Hz in the participant group of the first experiment). Note that matching the stimulation frequency to endogenous oscillations for obtaining entrainment could be particularly relevant for electrical stimulation, since the need for an exact match is magnified with weak stimulation forces (see Arnold tongues in entrainment models in, e.g., Pikovsky et al., 2003, but also Frohlich, 2015; Thut et al., 2017). This might not be the case for transcranial magnetic stimulation, a technique that allows for the induction of electric fields strong enough to trigger action potentials, and which has been shown to influence the balance of attention allocation even with fixed 10 Hz stimulation of parietal cortex (Romei et al., 2010; Ruzzoli and Soto-Faraco, 2014). In light of our findings, we therefore recommend to attempt an exact match between stimulation and natural frequencies when aiming to interact with oscillatory activity by tACS.

A second reason for our overall unsuccessful attempt to modulate attention bias could be differences in individual anatomy. Despite the fact that the simulation of the current distribution did suggest an electric field maximum within the right parietal cortex, the modeling was not informed by individual anatomy and therefore did not take into consideration individual skull thickness and gyri configuration. In light of this limitation, it is therefore possible that the amount and distribution of current reaching the brain varied across participants. Moreover, variations in individual anatomy will also influence the direction of the induced current flow (normal vs. tangential components) (De Berker et al., 2013). We believe that optimization of tES interventions will ideally require more detailed planning of stimulation montages before experimentation.

Stimulation outcome has been shown to depend on individual trait factors and the state of the cortex (Feurra et al., 2013; Neuling et al., 2013; Benwell et al., 2015; Alagapan et al., 2016). Here, we performed additional *post hoc* analysis on the entire sample in the attempt to identify possible trait factors co-varying with tES outcome (taking into account the measures we had available, i.e., PSE and curve width at baseline), but found no evidence of any influence. However, it is conceivable, that other factors, such as alpha power at baseline could have better predicted the stimulation effect, in line with studies showing that tACS can engage endogenous rhythms depending on the pre-existing power of the targeted oscillation (Neuling et al., 2013; Alagapan et al., 2016). Large-scale studies with large sample sizes (ideally multi-center) would be required to screen for possible predictors of outcome, and to test their validity.

Finally, it may be argued that the measure of spatial bias used here (PSE) is potentially confounded by response bias, because our participants were always asked to indicate which side of the stimulus appeared ‘shortest.’ This confound can be removed by alternating within participants across blocks in which they are requested to indicate the ‘shortest’ and ‘longest’ end of the line (Toraldo et al., 2004). However, several studies have previously shown baseline pseudoneglect to be a consistent bias in healthy, young individuals regardless of whether single and/or separate instructions were given (Jewell and McCourt, 2000; Schmitz et al., 2011; Benwell et al., 2013a,b, 2014a). For this reason, we believe that the leftward bias is not explained by a response bias. Additionally, potentially opposite α-tACS effects on perceptual and motor bias could explain the inconclusive results in the overall sample, but are unlikely to explain the significant effect of α-tACS in the first experiment.

## Conclusion

Taken together, our results highlight the need to adopt caution in interpreting tES results from single-studies when characterized by small sample sizes and arbitrary choices of stimulation parameters (frequency, electrode position, intensity of stimulation). Because these characteristics may potentially come with a lack of statistical power and/or an inadequate use of the stimulation itself, they limit the conclusions about the efficacy of the studied tES protocol. The specificity and efficacy of tES may be improved by taking into account individual differences in anatomy and other endogenous factors to optimize design parameters (e.g., stimulation frequencies, electrode montages, intensities etc.). However, we also believe that larger scale studies and direct replications are needed to establish the robustness and reliability of electrical stimulation effects, and to identify potential predictors of outcome.

## Author Contributions

DV, CB, and GT conceived the study and analyzed the data. DV, CB and MA collected the data. All authors wrote the manuscript.

## Conflict of Interest Statement

The authors declare that the research was conducted in the absence of any commercial or financial relationships that could be construed as a potential conflict of interest.

## Footnotes

1 www.simnibs.de
